# A designathon to co-create community-driven HIV self-testing services for Nigerian youth: findings from a participatory event

**DOI:** 10.1186/s12879-021-06212-6

**Published:** 2021-05-31

**Authors:** Kadija M. Tahlil, Chisom Obiezu-Umeh, Titi Gbajabiamila, Ucheoma Nwaozuru, David Oladele, Adesola Z. Musa, Ifeoma Idigbe, Jane Okwuzu, Agatha N. David, Tajudeen A. Bamidele, Collins O. Airhihenbuwa, Nora E. Rosenberg, Weiming Tang, Jason J. Ong, Donaldson F. Conserve, Juliet Iwelunmor, Oliver Ezechi, Joseph D. Tucker

**Affiliations:** 1 Department of Epidemiology, University of North Carolina at Chapel Hill, Chapel Hill, NC USA; 2 Department of Behavioral Science and Health Education, Saint Louis University, Saint Louis, MO USA; 3 Nigerian Institute of Medical Research, Lagos, Nigeria; 4 School of Public Health, Georgia State University, Atlanta, GA USA; 5 Department of Health Behavior, University of North Carolina at Chapel Hill, Chapel Hill, NC USA; 6 Dermatology Hospital, Southern Medical University, Guangzhou, China; 7 Faculty of Infectious and Tropical Diseases, London School of Hygiene and Tropical Medicine, London, UK; 8 Central Clinical School, Monash University, Melbourne, Australia; 9 Department of Health Promotion, Education, and Behavior, Arnold School of Public Health, The University of South Carolina, Columbia, SC USA; 10 Department of Medicine, University of North Carolina at Chapel Hill, Chapel Hill, NC 27599 USA

**Keywords:** Designathon, Crowdsourcing, Youth, Human immunodeficiency virus (HIV), Self-test, Nigeria

## Abstract

**Background:**

Youth are at high risk for HIV, but are often left out of designing interventions, including those focused on adolescents. We organized a designathon for Nigerian youth to develop HIV self-testing (HIVST) strategies for potential implementation in their local communities. A designathon is a problem-focused event where participants work together over a short period to create and present solutions to a judging panel.

**Methods:**

We organized a 72-h designathon for youth (14–24 years old) in Nigeria to design strategies to increase youth HIVST uptake. Proposals included details about HIVST kit service delivery, method of distribution, promotional strategy, and youth audience. Teams pitched their proposals to a diverse seven-member judging panel who scored proposals based on desirability, feasibility, potential impact and teamwork. We examined participants’ socio-demographic characteristics and summarized themes from their HIVST proposals.

**Results:**

Forty-two youth on 13 teams participated in the designathon. The median team size was 3 participants (IQR: 2–4). The median age was 22.5 years (IQR: 21–24), 66.7% were male, 47.4% completed tertiary education, and 50% lived in Lagos State. Themes from proposals included HIVST integration with other health services, digital marketing and distribution approaches, and engaging students. Judges identified seven teams with exceptional HIVST proposals and five teams were supported for further training.

**Conclusions:**

The designathon provided a structured method for incorporating youth ideas into HIV service delivery. This approach could differentiate HIV services to be more youth-friendly in Nigeria and other settings.

**Supplementary Information:**

The online version contains supplementary material available at 10.1186/s12879-021-06212-6.

## Background

Youth (age14–24 years) account for an estimated one-third of all new HIV infections globally [1]. Although youth are disproportionately affected by HIV, their contributions to the development of interventions designed for them have often been limited and sometimes tokenistic [2–9]. Youth typically contribute to research as participants, key informants, and assistants [10, 11]. While some studies have expanded youth engagement in HIV research by creating youth advisory boards (YABs), the extent of meaningful engagement varies [12–21]. Providing youth with opportunities to create solutions to health problems that affect them can enhance program implementation and build capacity for youth as co-creators [10, 22].

One strategy to meaningfully engage youth is crowdsourcing, a practice in which solutions to a social problem are solicited from multiple individuals and then shared with the public [23]. It provides space for people with diverse backgrounds to work together and share their solutions with local communities. One form of crowdsourcing is a designathon, a problem-solving event, typically held over a few days, in which participants work together to create, design, and present their proposed solutions to a panel of judges [24]. Designathons have been previously used to solve problems in education, technology, and public health. Designathons foster multi-disciplinary collaboration and can result in innovative solutions [24–26].

Responding to the need for youth engagement in HIV intervention design, we organized a designathon in Nigeria. The purpose was to develop ideas on how to promote HIV self-testing (HIVST) services among youth. This is part of the “Innovative Tools to Expand HIV Self-Testing” (ITEST) project, locally known as “4 Youth By Youth” [27]. HIVST is a process by which individuals collect their own oral fluid or blood specimen, conduct the HIV test, and interpret their results [28]. HIVST can help expand HIV testing services among those who have never tested before, such as many youth in Nigeria [28, 29]. The objective of this paper is to describe the designathon, summarize the resulting HIVST proposals, and discuss public health implications.

## Methods

The designathon was part of a multi-phase contest in which young people shared their ideas on how to promote HIVST among youth in Nigeria. This event was one component within the study’s broader youth participatory process and we frame its processes and outcomes within this multi-phase context. We describe the initial contest (phase I) preceding the designathon and the training program (phase III) succeeding the designathon in supplementary materials (see Additional file 1: Supplement 1). We describe the designathon (phase II) below.

Between March 29–31, 2019, we hosted the designathon in Lagos, where 13 youth teams selected to participate designed strategies to increase youth HIVST. The expected deliverables included a prototype of teams’ HIVST kit service delivery and a presentation of their idea to a panel of judges. We instructed teams to develop a community-based, youth-friendly model that would rely on modest payments. The price point was determined by earlier discrete choice experiments [30].

**Fig. 1:**
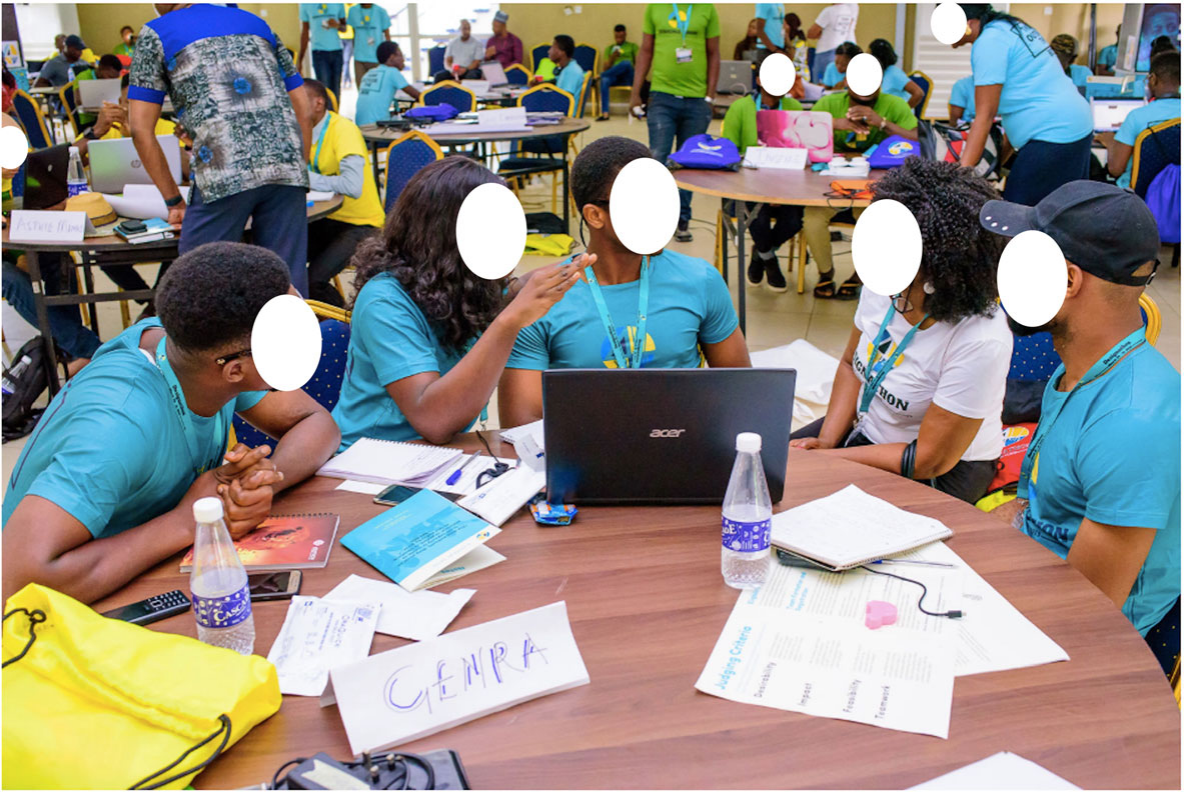
Designathon team discussing their project and receiving mentorship from member of the contest advisory panel

Day 1: We shared the purpose, rules, and expected deliverables of the contest to the teams. We familiarized participants with HIVST and briefed them about different testing kits in the Nigerian market. We scheduled panel discussions on challenges associated with standard HIV testing services and understanding the process of innovative problem-solving. Following the panel discussions, we asked participants to spend the remainder of the day in their teams to begin developing their HIVST proposals (Fig. 1).

Day 2: The teams participated in a presentation on how to effectively pitch their HIVST ideas. They continued working on their proposals, receiving tailored feedback (see Additional file 2: Supplement 2).

Day 3: We invited an independent panel of seven judges to listen to, review any support materials, ask questions and score teams’ HIVST proposals. The judges’ expertise included telecommunications, technology, product design, youth ambassadorship, and environmental sustainability. Each team, in turn, finalized their deliverables and presented their prototypes to the judges.

The judges evaluated proposals based on desirability, feasibility, impact, and teamwork. Desirability was defined as being appealing and meeting the needs of youth. Feasibility was defined as being easy to implement Nigeria. Impact was defined as having the potential to influence young people to self-test for HIV. Teamwork was defined as effectively working together. Each domain was scored on a three-point scale, with one being a low score, two being a medium score and three being a high score. The maximum score allotted to a team, from each judge, was 12 points. The top three teams were announced at the end of the designathon. The judges’ scores for each team’s HIVST proposal can be found in the supplementary materials (see Additional file 3: Supplement 3). We provided cash prizes to the top three teams: NGN 250,000 ($694) for first place, NGN 150,000 ($416) for second place, and NGN 50,000 ($138) for third place. We provided food, transportation, and accommodation to all participants.

### Data analysis

We performed descriptive statistics to characterize demographic data of participants. We summarized teams’ HIVST ideas with respect to their HIVST service delivery, method of distribution, promotional strategies, and youth audience.

## Results

### Participant characteristics

Forty-two young Nigerians were selected to participate in 13 teams (Table 1). The median team size was three participants (interquartile range: 2–4). The median age was 22.5 years (interquartile range: 21–24), 66.7% were male, 47.4% completed tertiary education, and 50% resided in Lagos State. Twenty-one (58.3%) participants were students and four (11.1%) were self-employed or entrepreneurs. Of 11 (30.6%) employed participants, five were members of the National Youth Service Corps (NYSC), two were nurses, two were employees at a private company, one was a laboratory assistant, and one worked in human resources.

**Table 1:** Characteristics of participants at the designathon: Nigeria 2019 (*N* = 42)

	n	%
**Age (Years)**		
18–21	14	33.3
22–24	28	66.7
**Gender**		
Female	14	33.3
Male	28	66.7
**Location (State)**		
Abuja	2	4.8
Cross River	4	9.5
Edo	1	2.4
Enugu	5	11.9
Kwara	1	2.4
Lagos	21	50
Ondo	2	4.8
Osun	1	2.4
Oyo	4	9.5
Rivers	1	2.4
**Highest Level of Education**		
Senior Secondary	15	39.5
Some Tertiary	5	13.2
Tertiary	18	47.4
Missing	4	
**Occupation**		
Employed	11	30.6
Self Employed/Entrepreneur	4	11.1
Student	21	58.3
Missing	6	

### Designathon pitches

The primary goal of pitches by teams was to propose solutions on how to increase HIVST among youth in Nigeria (Table 2). Their ideas varied across the HIVST service delivery, method of distribution, promotional strategies, and youth audience.

**Table 2:** HIV self-testing (HIVST) services proposed by the thirteen teams at the designathon: Nigeria 2019

Rank	Team Characteristics	Members	State	HIVST Project Proposal	Digital Approaches	Youth Audience	Score^**b**^
1	Public Health Professionals	2	Oyo	Develop a website that will allow individuals to purchase HIVST and other STI kits. The website will provide access to trained volunteers, instructions on how to self-test, and information on post-testing services. This team will utilize peer-to-peer referral to increase distribution of kits.	None specified.	Rural adolescent girls NYSC members	71
2	Tertiary Students	4	Enugu	HIVST kits will be distributed through various offline retailers and social media. The kits will be equipped with a code that individuals will need to gain access to the team’ social media app. The social media app wil allow individuals to report their results and find their nearest youth-friendly clinic.	Create a social media app for young people who have purchased HIVST kits. Conduct an online marketing campaign through social media. Distribute kits through WhatsApp, Facebook, Twitter and Instagram.	Tertiary students Young people on social media	70
3^a^	NYSC Members Entrepreneur	5	Lagos	HIVST kits will be packaged with self care products (condoms, sanitary pads, soaps). Young people will buy the kit online, at a pharmacy, gym or retail outlet. Upon completion of the self-test, individuals can submit their results to a study phone number and receive post-test services.	Conduct an online marketing campaign through social media.		62
4	Tertiary Students	5	Lagos	HIVST kits will be packaged with self care products (condoms and lubricants). The kits will be distributed through online and offline platforms. In additon to online marketing, the team will rely on peer-to-peer referral to increase distribution of kits.	Conduct an online marketing campaign through social media.	Out-of-school AYPs FSWs MSM	63
5	Tertiary Students	2	Lagos	Develop a mobile/web app that will allow individuals to purchase HIVST kits, record their test results, obtain information on HIV, and access post-testing services. The team will utilize peer-to-peer referral to increase distribution of kits.	Conduct an online marketing campaign through social media.	Secondary and tertiary students	61
6	Tertiary Students	2	Ondo	HIVST kits will be packaged with nutritional supplements. Students will buy the kits at pharmacies, or through campus ambassadors. Upon completion of the self-test, students can access offline or online post-test services.	Conduct an online marketing campaign through social media.	Tertiary students	51
7	Tertiary Students	2	Oyo	HIVST kits will distributed through recharge card vendors and SIM registration centers.	Conduct an online marketing campaign through social media.		51
8	Tertiary Students Human Resources Professional	3	Lagos	HIVST kits will be distributed through vending machines. Young people will provide their phone numbers during the purchase and receive a link to download the team’s app, which will give them access to various health services.	None specified.	Tertiary students	49
9	Tertiary Students Entrepreneur	5	Edo Lagos	HIVST and other STI kits will be packaged with self care products (condoms, sanitary pads, soaps). The kits will be equipped with a code that individuals can use to text the team for follow-up.	None specified.		47
10	NYSC Members	2	Lagos	Develop a website that will direct individuals to their nearest health facility to collect HIVST kits and receive pre-counseling services. The kits will contain an instruction manual on how to use the kit and information on the appropriate post-testing services.	None specified.	Tertiary students NYSC members	47
11	Tertiary Student Nurses Laboratory Teaching Assistant	4	Kwara Osun Enugu Rivers	Develop a mobile app that will allow young people to purchase HIVST and other STI kits. Individuals will use the mobile app to access post-test counseling services, get information about HIV risk-reduction strategies, and be referred for confirmatory testing and care.	None specified.		47
12	Private Company Employees	2	Abuja FCT	Develop a mobile app that will allow Individuals to play an interactive game. Throughout the game, individuals will be provided with information about HIVST. At the end of the game, individuals will be shown a toll-free number they can call to acquire HIVST kits and information about HIV and other STDs.	None specified.		45
13	Tertiary Students Self-employed Professionals	4	Cross River	Develop a website that will direct individuals to their nearest health facility to collect HIVST kits. Individuals with no internet access can use their mobile phones to dial a short code and get information about where to obtain HIVST kits.	Conduct an online marketing campaign through social media.		42

Tertiary students in Nigeria are individuals who attend universities, polytechnics, monotechnics, or colleges of education

*HIVST* HIV self-testing, *NYSC* National Youth Service Corps, *AYPs* Adolescent and Young People, *FSWs* Female Sex Workers, *MSM* Men who have sex with men

^a^This team scored lower than the fourth place team, but the judges deliberated and decided to elevate this team to third place because their concept was more attractive and could better appeal to young people

^b^Scores were rounded to the nearest whole number

### HIVST service delivery

Five teams proposed delivering HIVST kits with other STI testing or health products. Three of these teams focused on creating a bundle product that combined HIVST with self-care products (e.g. condoms, lubricants, panty liners), one of which finished in third place. Of the remaining two teams, one pitched selling two HIVST kits in one set to reduce the purchasing cost for young people and another proposed complementing HIVST with nutritional supplements.

### Method of distribution

Six teams proposed an online platform to sell kits. These online platforms included websites, online retail stores, and mobile phone applications. Among the six teams with an online platform, three also had an offline distribution strategy, such as placing their kits at pharmacies, retail outlets, or gyms to reach young people who may not have access to phones or the internet. Six teams proposed to distribute their kits solely offline. Among these six teams, two developed creative methods to distribute their kits. One team proposed to stock vending machines with HIVST kits in areas with more youth and another team would partner with mobile phone card vendors.

### Promotional strategies

Seven teams planned to use social media (WhatsApp, Facebook, Instagram, and Twitter) to conduct online marketing campaigns for their kits. One team, which finished in second place, developed their own social media application where youth could report their test results and find post-test HIV services. Three teams described a peer referral system to enhance HIVST uptake, of which two incentivized youth for peer referrals.

### Youth audience

Five teams planned to focus on youth in school. Four teams focused on tertiary students and one team focused on both secondary and tertiary students. One team planned to engage hard-to-reach populations such as out-of-school youth, female sex workers, and men who have sex with men. One team proposed to recruit rural adolescent girls and members of the National Youth Service Corps (NYSC), a mandatory service program for Nigerian youth. This team, which finished in first place, proposed leveraging youth service networks to distribute kits and encourage HIVST.

Designathon proposals shared the following characteristics: educating youth on their health status and where to obtain appropriate services; offering care relevant to the needs of youth; and providing the opportunity for youth to participate in health service delivery. Furthermore, the teams provided youth with alternatives to clinical settings to access HIV and services. Moreover, all teams underscored the importance of maintaining young peoples’ rights to privacy and confidentiality. The teams also proposed to partner with institutions (e.g. schools, local community centers) where youth frequent to advertise and distribute their kits.

## Discussion

We organized a designathon in which we crowdsourced ideas on how to increase HIVST among Nigerian youth. This designathon provided an opportunity for young Nigerians to collaborate and develop strategies. We extend the literature by using a designathon methodology, allowing robust input from youth, and providing structured feedback and mentorship.

The designathon generated proposals that were determined by a judging panel to be potentially feasible, desirable, and impactful. The participants’ diverse backgrounds and expertise may have helped in the development of promising proposals. Youth with different perspectives can draw from their experiences to develop potentially feasible interventions. This insight is consistent with a previous designathon in the United States that brought together diverse individuals [25]. The high quality of submissions may also have been related to their knowledge of local health services and youth preferences [22].

The designathon engaged local youth and allowed them to meaningfully contribute. Youth participants led the design of HIVST interventions, which is rare in health research [31, 32]. There are implications for allowing youth to design solutions to health problems. Although the designathon was open to youth age 14–24 years, individuals selected to participate were all 18–24 years. Thus, older adolescents were more successful in presenting promising HIVST ideas. Moreover, participants were aware selected finalists would be allowed to not only design an HIVST strategy but implement it in their local areas. It is possible that this designathon encouraged a sense of community ownership among participants, which has been observed in a previous HIV-related designathon [24].

Several proposals integrated HIVST with sexual/reproductive health services. This finding is consistent with larger literature underlining the importance of integrating HIV testing services and sexual/reproductive health services [33–38]. In these other contexts, HIV testing service had been embedded within family planning, STI testing, and sexual health counseling. These studies demonstrate the integration of HIV services with other health services increases testing acceptability and health service use, improving the quality of care [33–37]. Given that many youth at risk for HIV also need other services, integrated HIV care may be an effective approach to reach young people.

Our study has several limitations. First, the judges selected HIVST proposals that had the potential to be feasible, desirable, and impactful. Judges did not have outcome data on these domains when judging, but pilots are now underway. Second, designathons are typically held over a few days, much shorter than would allow them to develop a comprehensive HIVST plan. However, following our designathon, selected finalists were then invited to a four-week training program to build research and entrepreneurial skills and implement and manage their programs. Finally, the designathon is resource-intensive, which can be challenging in resource-limited settings. To minimize costs, we leveraged local resources, such as staffing from the Nigerian implementing team and passionate volunteers to serve as mentors.

The designathon contained novel features, which has implications for public health research and policy. First, this event allowed youth to lead in the development of interventions that could benefit them, which is rare in many HIV programs for youth in low and middle-income countries. Designathons may provide an opportunity for youth to take health ownership and lead health research. Furthermore, results from these teams’ proposals can help HIV testing services be more responsive to the unique needs of youth. Further implementation science is needed to evaluate the effectiveness of the proposed ideas.

## Conclusions

We described the processes and outcomes of the first health-related designathon in Nigeria that focused on improving HIVST among youth. Youth-led development of HIVST strategies offers promising solutions to expand HIVST among young people in Nigeria.

## Supplementary Information

**Additional file 1: Supplement 1.** Timeline of study events: Nigeria 2019.

**Additional file 2: Supplement 2.** Photographs from the designathon: Nigeria 2019.

**Additional file 3: Supplement 3.** Judges' scores for all teams at the designathon: Nigeria 2019*.

## Data Availability

The datasets used and/or analyzed during the current study are available from the corresponding author on reasonable request.

## Abbreviations

AIDS: Acquired Immunodeficiency Syndrome
HIV: Human Immunodeficiency Virus
HIVST: HIV Self-testing
ITEST: Innovative Tools to Expand HIV Self-Testing
NGN: Nigerian Naira
NYSC: National Youth Service Corps
STI: Sexually Transmitted Infection
YAB: Youth Advisory Board
