# Supplementary material for: A designathon to co-create community-driven HIV self-testing services for Nigerian youth: findings from a participatory event
Source: BMC Infect Dis. 2021 May 31;21:505. doi: 10.1186/s12879-021-06212-6 (PMC8166032; doi:10.1186/s12879-021-06212-6)
Supplement: Supplementary file 1 — Additional file 1: Supplement 1. Timeline of study events: Nigeria 2019. [file 12879_2021_6212_MOESM1_ESM.docx]

Supplement 1. Timeline of study events: Nigeria 2019

*Organization*

A contest advisory panel was created to oversee the logistics and overall organization of designathon activities. The panel consisted of a diverse set of 18 experts and professionals from the fields of design thinking, public health, communications, and non-profit organizations. The purpose of the panel was to provide mentorship and guidance to the youth participants as they developed their HIVST proposals and to evaluate the entries submitted. The panel met periodically between October 2018 – March 2019 to prepare for the designathon. The designathon was part of a multi-phase contest and a broader interventional development process. We describe the initial contest (phase I) preceding the designathon, the preparation activities prior to the designathon (phase II), and the training program (phase III) succeeding the designathon below.

*Phase One: Open Individual Crowdsourcing Contest*

Between October 15 – November 25, 2018, we conducted a crowdsourcing contest in which Nigerians aged 10 – 24 years submitted their ideas (written, photos, or videos) addressing the following contest question: “How will you promote HIV self-testing among young people in Nigeria” [27]? We advertised the contest through blogs, social media, print communication, and visits to secondary schools. A more detailed description of this process is published elsewhere [27]. Of the 769 valid submissions, we invited individuals from the top 42 submissions to the World AIDS Day Event on December 1, 2018 in Lagos to pitch their ideas to a six-judge panel. The panel of judges consisted of the President of the Nigeria Youth Network on HIV/AIDS, a 4 Youth By Youth ambassador, a program manager with the Lagos State AIDS Control Agency, a public health professor, a communications expert, and an independent clinician. The participants with the top five ideas from the contest were invited to join the designathon. Furthermore, the ideas generated from the contest informed the contest question for the designathon.

*Phase Two: Pre-Designathon Process*

From January 25 – March 10, 2019, we solicited submissions for the designathon using similar promotion methods as the contest in phase I. The eligibility criteria were that applicants were between the ages of 14-24 years, resided in Nigeria, and part of a team of two to five members. Of the 127 entries that were received, 75 met the eligibility criteria. Of the remaining 52 entries that were ineligible, three were due to incomplete application forms and 49 were due to individual applications that did not meet the team requirement. Four members of the contest advisory panel reviewed the 75 eligible entries and selected teams that answered the designathon problem statement: “How might we encourage self-testing for HIV and other sexually transmitted diseases among young people (ages 14-24) in Nigeria in a way that is low cost, accessible, youth-friendly and confidential?” The contest advisory panel determined there were 20 eligible entries that adequately addressed the designathon problem statement. These 20 entries were reviewed and scored by four members of the contest advisory panel and two 4 Youth By Youth ambassadors. Entries were evaluated on a three-point scale based on the desirability, feasibility, and impact of their HIVST proposals. The top eight teams from phase II were invited to join the five teams from phase I for the designathon (N=13 teams total).

*Phase Three: Training Program*

Between May 6 – May 30, 2019, we conducted a four-week training program with five selected teams from the designathon. The purpose of the program was to have youth participants continue the development of their designathon proposal into a comprehensive HIVST service through further cultivation of their research and entrepreneurial skills. A more detailed description of this process is published elsewhere [38]. The outcome of the training program was five HIVST services ready for pilot implementation in different communities across Nigeria.
