# Supplementary figures and images for: A designathon to co-create community-driven HIV self-testing services for Nigerian youth: findings from a participatory event
Source: BMC Infect Dis. 2021 May 31;21:505. doi: 10.1186/s12879-021-06212-6 (PMC8166032; doi:10.1186/s12879-021-06212-6)

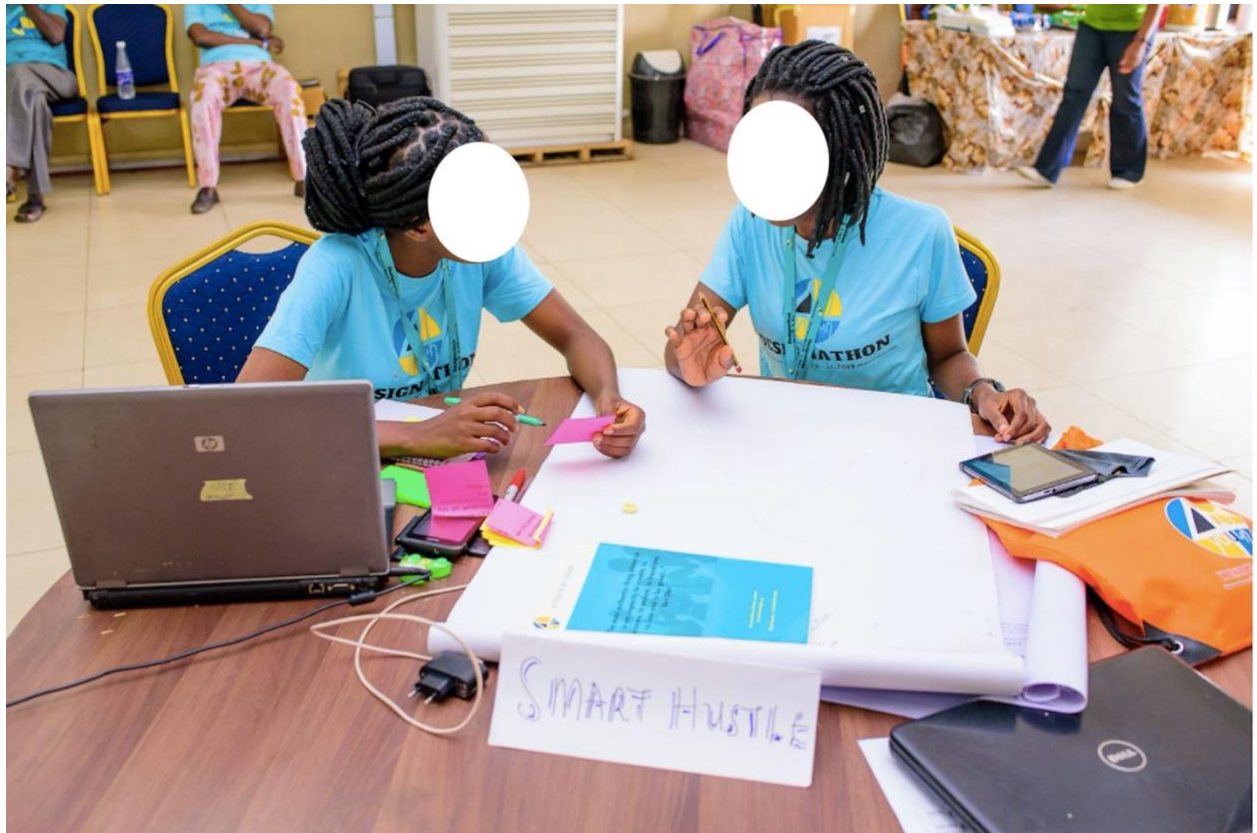

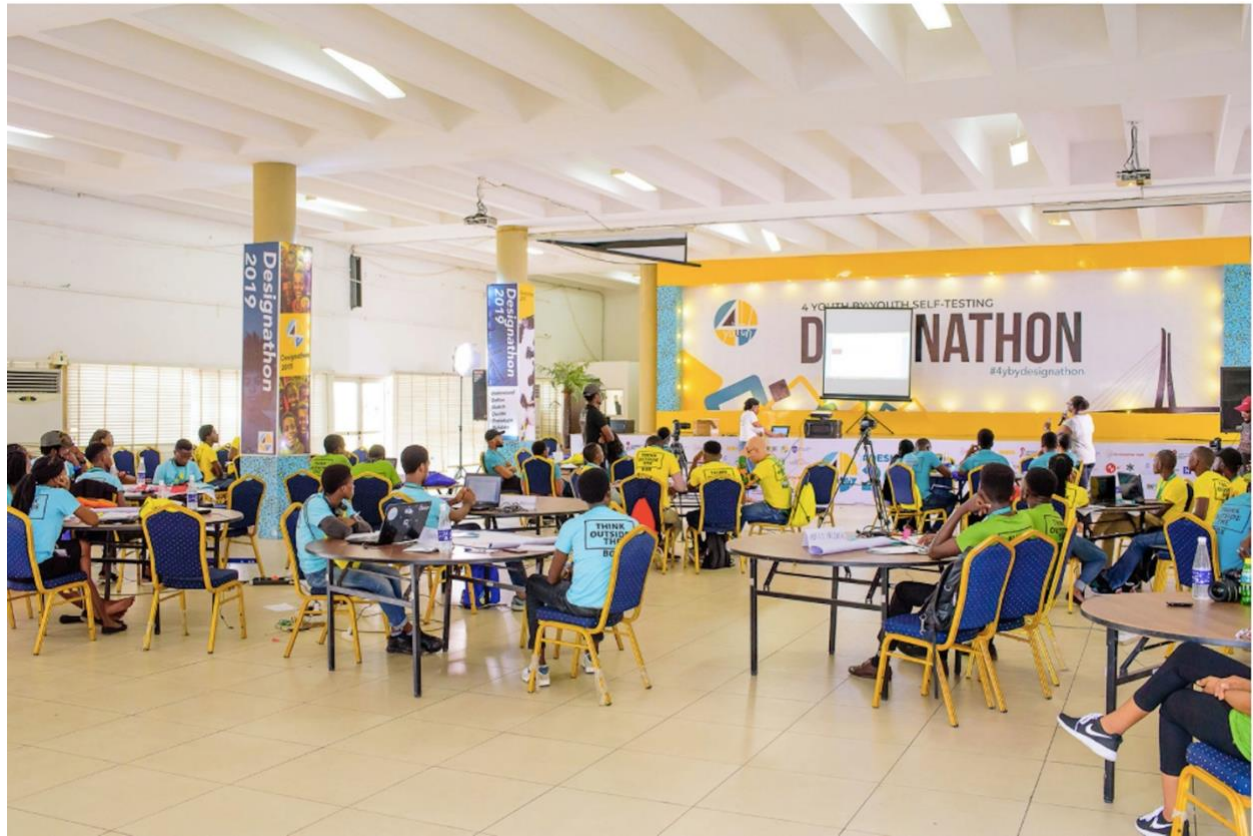

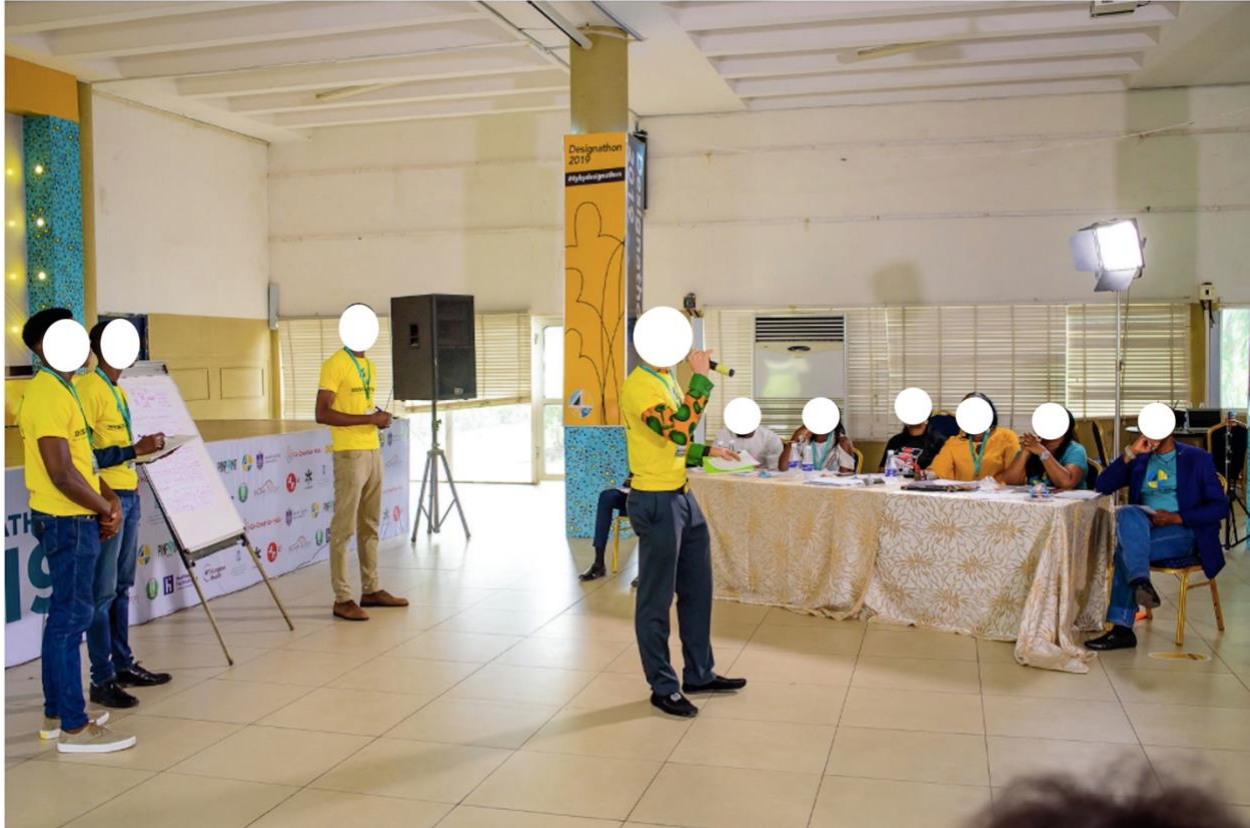

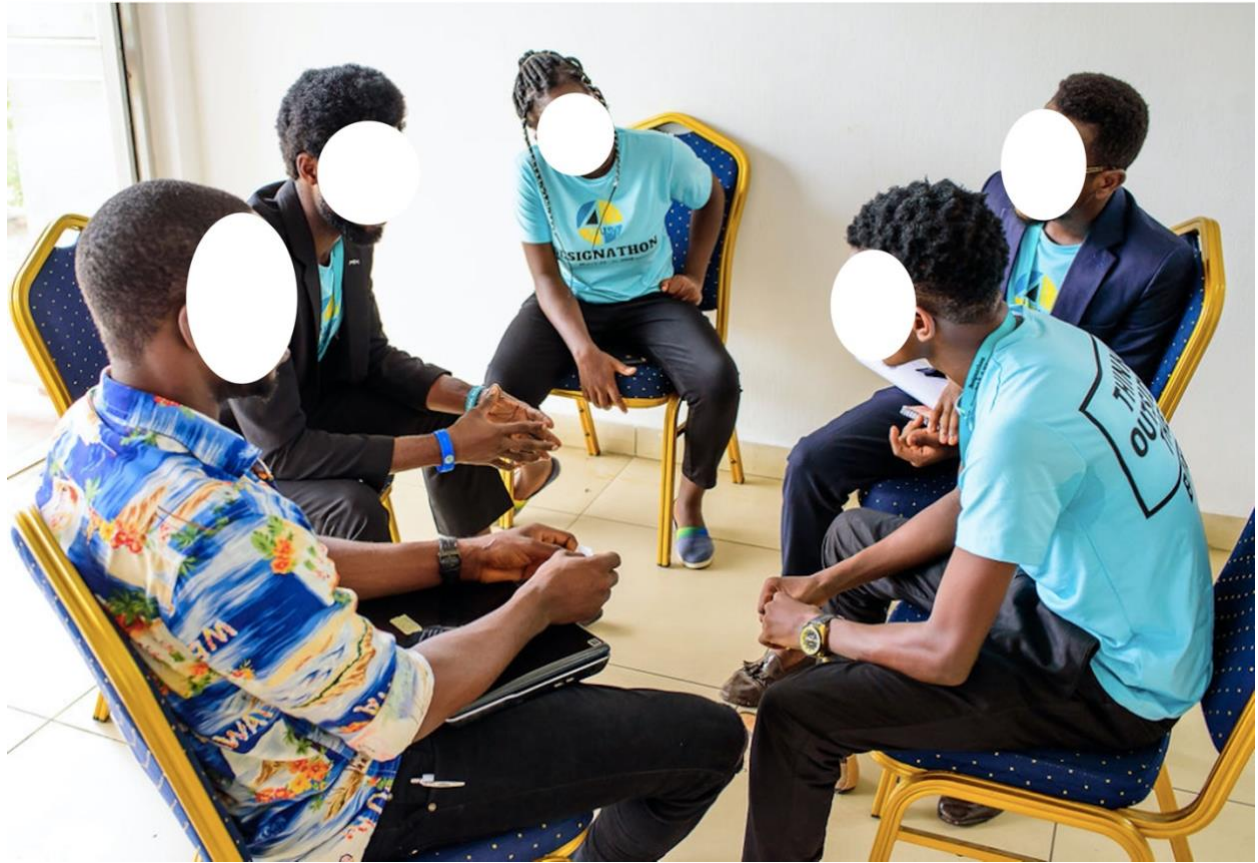

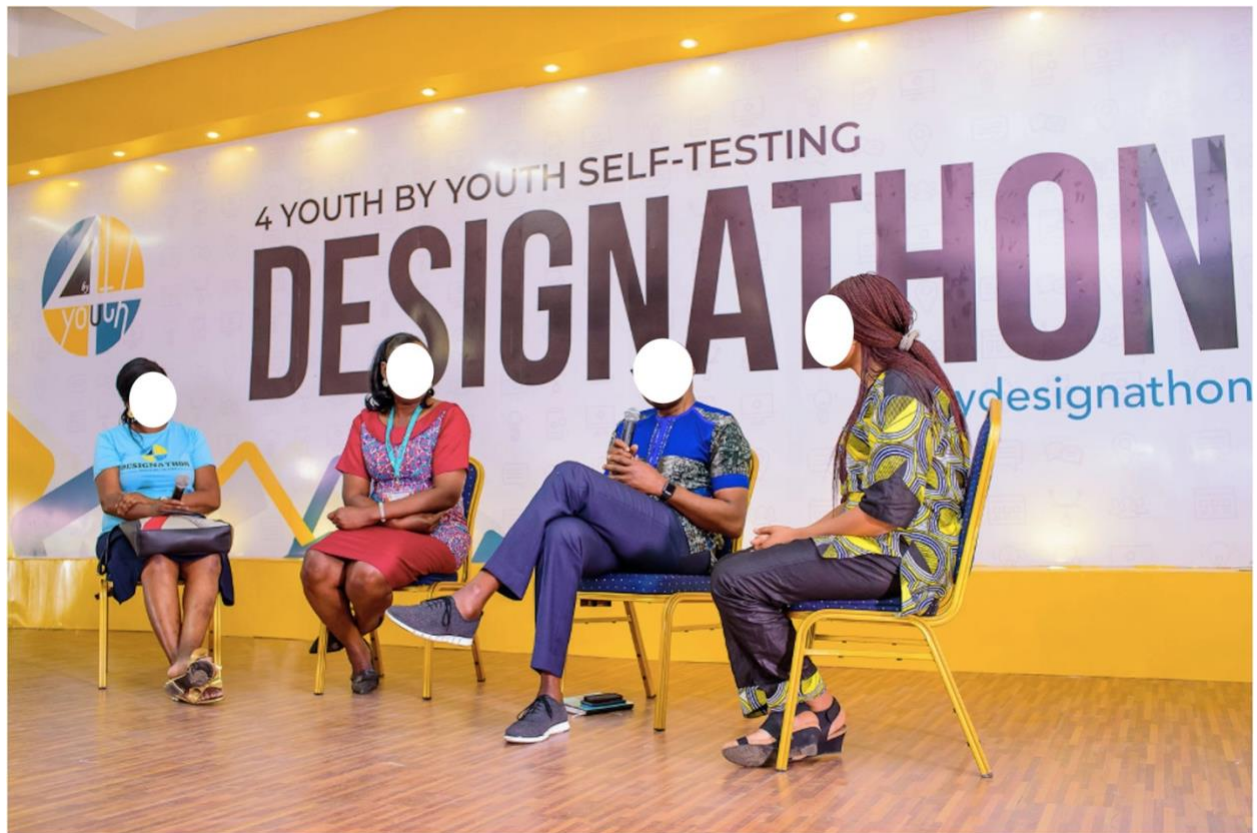

Supplement: Supplementary file 2 — Additional file 2: Supplement 2. Photographs from the designathon: Nigeria 2019. [file 12879_2021_6212_MOESM2_ESM.pdf]
